# Changes in Corticospinal Tract Consistency after Dry Needling in a Stroke Patient

**DOI:** 10.1155/2024/5115313

**Published:** 2024-09-14

**Authors:** Masoome Ebrahimzadeh, Noureddin Nakhostin Ansari, Iraj Abdollahi, Behnam Akhbari, Jan Dommerholt

**Affiliations:** ^1^ School of Medicine Hormozgan University of Medical Sciences, Bandar Abbas, Iran; ^2^ Department of Physiotherapy University of Social Welfare and Rehabilitation Sciences, Tehran, Iran; ^3^ Department of Physiotherapy School of Rehabilitation Tehran University of Medical Sciences, Tehran, Iran; ^4^ Research Center for War-affected People Tehran University of Medical Sciences, Tehran, Iran; ^5^ Bethesda Physiocare, Bethesda, MD, USA; ^6^ Myopain Seminars, Bethesda, MD, USA; ^7^ Department of Physical Therapy and Rehabilitation Science School of Medicine University of Maryland, Baltimore, MD 21201, USA

## Abstract

**Background:**

Dry needling (DN) is a technique employed to mitigate spasticity and enhance functionality in stroke patients. We report the impact of DN on both corticospinal tract (CST) consistency and wrist flexors spasticity of an individual affected by stroke.

**Case:**

The participant was a 57-year-old male who had experienced an ischemic stroke 9 months prior. The primary outcome measures included fractional anisotropy (FA), asymmetry FA (aFA), ratio FA (rFA), and Modified Modified Ashworth Scale (MMAS). Additionally, secondary outcomes encompassed wrist extension range of motion (ROM) and performance in the box and block test (BBT). These measurements were taken both before and after the administration of DN treatment.

**Results:**

After the application of DN, the mean FA of the ipsilesional CST increased from 0.35 to 0.39, concomitantly with a decline in aFA from 0.18 to 0.13. Notably, the rFA exhibited a pre-DN value of 0.69, which subsequently rose to 0.76 post-DN. Moreover, a significant reduction in MMAS scores was detected, from a score of “3” prior to DN application to a post-DN score of “1”. In terms of wrist mobility, both active and passive extension ROM exhibited favorable improvements, with an increase of 12° for active extension and 16° for passive extension. Furthermore, there was a substantial improvement in the BBT score, an indicator of manual dexterity, ascending from 12 to 24.

**Conclusion:**

Enhancements in CST consistency suggest it as a potential mechanism contributing to the observed improvements following DN in this stroke case.

## 1. Introduction

Stroke is the second-leading cause of death and the third-leading cause of death and disability in the world [1]. According to a recent global burden disease study, the stroke is ranked the first among the top ten conditions with the highest age-standardised disability-adjusted life-years (DALYs) in 2021 [2]. Spasticity post-stroke is a common motor disorder that contributes to functional impairment and decreased quality of life. The prevalence of spasticity in Iran is reported between 17 and 25% during the first 3 months after a first-time stroke [3].

Spasticity interferes with active movement due to increased excitability of the stretch reflex [4]. The corticospinal tract (CST) is a major pathway for skilled voluntary movements that is injured after stroke [5]. There is a significant correlation between CST remodeling after a stroke and spasticity reduction [6]. Dry needling (DN) is a technique used to control muscle spasticity. Previous studies showed improvements in muscle spasticity, active movements, motor neuron excitability, and brain activity after DN in patients with stroke [7–11]. We hypothesized that improvements are associated to the neuromodulating effects of DN on CST consistency. To the best of our knowledge, this case study represents the first attempt to quantify the influence of DN on CST consistency through diffusion tensor imaging (DTI) in a patient with stroke. The improvement in CST consistency suggests its potential role as a mechanism influenced by DN. Here, we present a patient with stroke and spasticity and report changes in CST consistency using DTI after DN intervention.

## 2. Case Presentation

The subject was a 57-year-old man suffering from chronic ischemic stroke with right hemiplegia and spasticity for 9 months duration. He was able to walk independently. He had no comorbidities such as cardiovascular disease, diabetes, or hypertension. Before the initiation of DN treatment, he had received rehabilitation programs and medications.

Wrist flexors spasticity on the affected side was assessed using the Persian version of Modified Modified Ashworth Scale (MMAS), in which the spasticity is graded from “0” (no increase in muscle tone) to “4” (affected part rigid in flexion or extension) [12]. A standard goniometer was used for measuring active and passive range of motion (ROM) of the wrist extension. Hand dexterity was assessed by box and block test (BBT) [13].

Diffusion Tensor Imaging (DTI) is a magnetic resonance imaging (MRI) method that is used to measure consistency of the CST. MRI images were acquired on a Siemens 3.0 Tesla scanner (Prisma, Erlangen, Germany), with 20-channel head coil at the Iranian National Brain Mapping Lab (NBML). Constrained Spherical Deconvolution (CSD) method was used to CST tractography [14]. Fractional anisotropy (FA) that reflects directionality of molecular motion in white matter was extracted in three regions of interest (ROIs) (pyramids, cerebral peduncles, posterior limbs of internal capsule) and the entire CST [15]. The assessment encompassed both the asymmetry of the CST (FA_contralesional_ − FA_ipsilesional_)/(FA_contralesional_ + FA_ipsilesional_), as well as the ratio FA (rFA) in the CST (FA_ipsilesional_/FA_contralesional_) [16]. All measurements were assessed before and after DN treatment.

After the baseline assessment, the flexor carpi radialis (FCR) and flexor carpi ulnaris (FCU) on the affected side were dry needled for three sessions, with 48 hours interval between sessions using a sterile, stainless, and disposable needle with the size of (0.25 × 0.30; DongBangAcuPrime Ltd., Korea). Each muscle was needled using fast-in and fast-out technique for 1 minute. The approximately motor points of the muscles were needled [17].

The MMAS score for wrist flexors, which was “3” prior to DN, exhibited improvement and was reduced to “1” following the DN intervention. Notably, both active and passive wrist extension ROM showed positive changes, with enhancements of 12° and 16°, respectively, after DN. The results from the BBT showed improvements in hand dexterity, from a pre-DN score of “12” to a post-DN score of “24” (Table 1).

Baseline FA value within the three ROIs and the ipsilesional CST were notably lower compared to the contralateral side. Following the DN treatment, post-DTI findings revealed elevated FA values for both the ipsilesional and contralateral sides (Table 2).

**Figure 1:**
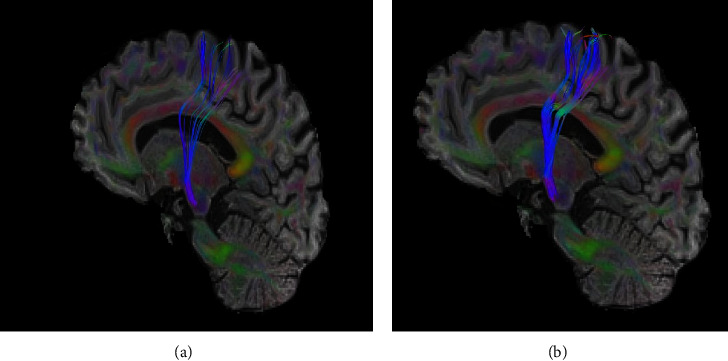
Sagittal view of the ipsilesional corticospinal tract (CST) pre (a) and post (b) dry needling (DN). Note that the fractional anisotropy as an indicator of CST connectivity increased after DN (blue color).

Following DN treatment, the mean FA of ipsilesional CST increased from 0.35 at baseline to 0.39. The baseline FA asymmetry, measured at 0.18, decreased to 0.13 after the application of DN. The pre-DN rFA was 0.69, which subsequently elevated to 0.76 post-DN intervention. Moreover, the FA value at the pyramid site was 0.26 at baseline increased to 0.28 after DN treatment.  Figure 1 shows the improvement in CST connectivity after DN.

## 3. Discussion

In this case study across three DN sessions, there was a significant increase in CST consistency, coincided with improvements in wrist flexor spasticity, active and passive wrist extension ROM, as well as manual dexterity as assessed by hand dexterity test of BBT.

Based on the DTI findings, the lower FA value observed in the ipsilesional CST prior to DN, in contrast to the contralesional CST, implies a compromised axonal connectivity and structural integrity in the ipsilesional CST. The subsequent elevation of FA in the three RIOs and the entirety of the CST, accompanied by the reduction in aFA post-DN, signify the effectiveness of DN in improving CST connectivity in this patient with stroke. These FA changes may indicate positive developments in axonal packing density, axonal diameter, myelination, neurite density, and the distribution of orientation within the CST [16].

Utilizing FA asymmetry to compare both sides of the CST and rFA provide valuable insights into CST structural enhancements. In this case, the reduction in FA asymmetry and increases in rFA subsequent to DN treatment suggest an improved physiological equilibrium between the ipsilesional and contralesional areas, signifying improved CST integrity and functionality [16]. Recently, two case studies reported positive impacts of DN on brain activity and brain network alterations post-stroke. These studies employed functional MRI and electroencephalogram techniques to demonstrate the favorable outcomes resulting from DN [8, 18]. Moreover, previous studies showed improvements of alpha motor neuron excitability following DN in patients after stroke [7]. Collectively, these findings underscore the neuromodulatory effects of DN, which have led to notable improvements in both clinical and the neurophysiological measures after stroke.

The reduction of two points in the spasticity grade of MMAS following DN in this stroke case surpasses the reported minimally clinically important changes (MCIC) of 0.48 and 0.76 documented for upper extremity muscles (elbow flexor, wrist flexor, and finger flexor) [19]. The improvements in spasticity, as evidenced by the decrease in MMAS grade, coupled with enhancements in both passive and active wrist extension, are consistent with previous reports in the literature [7]. Improvements in both wrist active and passive extension may be attributed to the reduction in muscle spasticity and enhancements in CST consistency.

This individual with stroke displayed a noteworthy increase of 12 points in the BBT following DN treatment. This improvement surpasses the established MCIC threshold of 5.5, signifying a clinically meaningful improvement in hand dexterity and function facilitated by the DN intervention. This improvement in BBT performance and overall hand function could potentially be attributed to several factors, including a reduction in spasticity, enhancements in wrist active extension, and improved consistency within the CST [17, 20].

### 3.1. Conclusion

A series of three sessions of DN yielded improvements in CST connectivity, wrist flexor spasticity, wrist active and passive extension, as well as hand dexterity in a patient with chronic stroke. DN as an inexpensive method with easy application and remodeling effects on CST can be considered as a good therapeutic strategy for control of muscle spasticity in stroke patients. To verify these findings, future studies with larger sample sizes and control groups are recommended, allowing for a more comprehensive understanding of DN's effects.

## Figures and Tables

**Table 1:** Clinical outcomes pre- and post-dry needling treatment.

Variables	Pre	Post
MMAS score	3	1
Wrist active extension ROM (degree)	28	40
Wrist passive extension ROM (degree)	49	65
Box and block Test(number)	12	24

*Note.* MMAS, Modified Modified Ashworth Scale; ROM, range of motion.

**Table 2:** Fractional anisotropy values, pre- and post-dry needling.

Region of interest	Ipsilesional FA		Contralesional FA	
	Pre	Post	Pre	Post
Corticospinal tract	0.35	0.39	0.50	0.51
Cerebral peduncle	0.26	0.36	0.42	0.44
Posterior internal capsule	0.57	0.58	0.59	0.59

FA, fractional anisotropy.

## Data Availability

Data sharing not applicable to this article as no datasets were generated or analyzed during the current study.
